# Endoplasmic reticulum stress signaling and chemotherapy resistance in solid cancers

**DOI:** 10.1038/oncsis.2017.72

**Published:** 2017-08-28

**Authors:** T Avril, E Vauléon, E Chevet

**Affiliations:** 1 INSERM U1242, ‘Chemistry, Oncogenesis, Stress, Signaling’, Université de Rennes 1, Rennes, France; 2 Centre de Lutte contre le Cancer Eugène Marquis, Rennes, France

## Abstract

The unfolded protein response (UPR) is an adaptive cellular program used by eukaryotic cells to cope with protein misfolding stress. During tumor development, cancer cells are facing intrinsic (oncogene activation) and extrinsic (limiting nutrient or oxygen supply) challenges, with which they must cope to survive. Moreover, chemotherapy represents an additional extrinsic challenge that cancer cells are facing and to which they adapt in the case of resistance. As of today, resistance to chemotherapy and targeted therapies is one of the important issues that oncologists have to deal with for treating cancer patients. In this review, we first describe the key molecular mechanisms controlling the UPR and their implication in solid cancers. Then, we review the literature that connects cancer chemotherapy resistance mechanisms and activation of the UPR. Finally, we discuss the possible applications of targeting the UPR to bypass drug resistance.

## Introduction

The endoplasmic reticulum (ER) is the first intracellular compartment of the secretory pathway. It regulates calcium homeostasis, lipid biosynthesis and protein productive folding and quality control. About one-third of all the proteins transit through the ER^1, 2, 3^ towards their final cellular or extracellular location. The synthesis of these proteins occurs on the cytosolic side of the ER and productive protein folding is orchestrated by elaborated ER-resident molecular machines involving chaperones, foldases and quality control proteins. These molecular machines ensure protein biogenesis from their nascent form to their ER exportable form.^4^ However, in the course of this process, a significant proportion of proteins is not properly folded and fails ER protein quality control criteria.^5^ These misfolded proteins are therefore addressed to the ER-associated degradation (ERAD) system that targets them to the cytosol for ubiquitinylation and proteasomal degradation.^1^ If the ER faces an important protein folding demand or sees its folding and degradation capacity attenuated, is needed, ER capacity to handle protein biogenesis are overwhelmed, thereby leading to an accumulation of improperly folded proteins in this compartment and to a situation called ER stress. ER stress leads to the activation of an adaptive response, named the unfolded protein response (UPR) that aims at (i) limiting misfolded proteins accumulation in the ER by transiently attenuating protein translation; (ii) augmenting the ER folding capacity by increasing the transcription of ER-resident chaperones proteins; (iii) enhancing protein clearance from the ER by increasing its degradation capacity. If the ER stress persists, the UPR triggers cell death.^6, 7^

During cancer genesis, an acute demand of protein synthesis is needed to support different cellular functions such as tumor proliferation, migration and differentiation, often driven by oncogenic activation.^3^ Tumor microenvironment might also provide limited tumor growth/development conditions because of important tumor oxygen and nutrient demands and inadequate vascularization. Therefore, cancer cells have to adapt to such a selective milieu with hypoxia, pH variation and nutrient deprivation that leads to cellular stress,^6, 8, 9, 10^ by activating a range of cellular stress-response pathways including the UPR that will be described in the first part of this review.

Chemotherapy represents an additional source of cellular stress for cancer cells. Indeed, antitumor drugs emphasize the microenvironmental stress acting on the selection of drug-resistant cancer cells.^11^ Resistance to chemotherapy is a principal problem in treating the most commonly seen solid tumors. Chemotherapy efficacy is indeed exposed to the multiple intrinsic and acquired resistance mechanisms developed by tumor cells that will be presented in the second part of this review. Furthermore, we will discuss the involvement of the ER stress-induced UPR to anticancer drug resistance. Understanding the UPR mechanisms associated with cancer drug resistance will provide insights to open new therapeutic avenues in which the association of standard chemotherapy with drugs targeting the UPR could overtake cancer drug resistance.

## UPR molecular mechanisms and their functions in cancers: the basics

The UPR is crucial for cells to adapt their ER folding capacity to selective conditions as such nutrients and oxygen privation.^1^ However, if environment-triggered ER stress cannot be resolved, prolonged UPR activation initiates cell death mechanisms. In this section, we will present the molecular actors of the UPR and describe its involvement in cancers.

### UPR sensors and their downstream pathways

**Figure 1:**
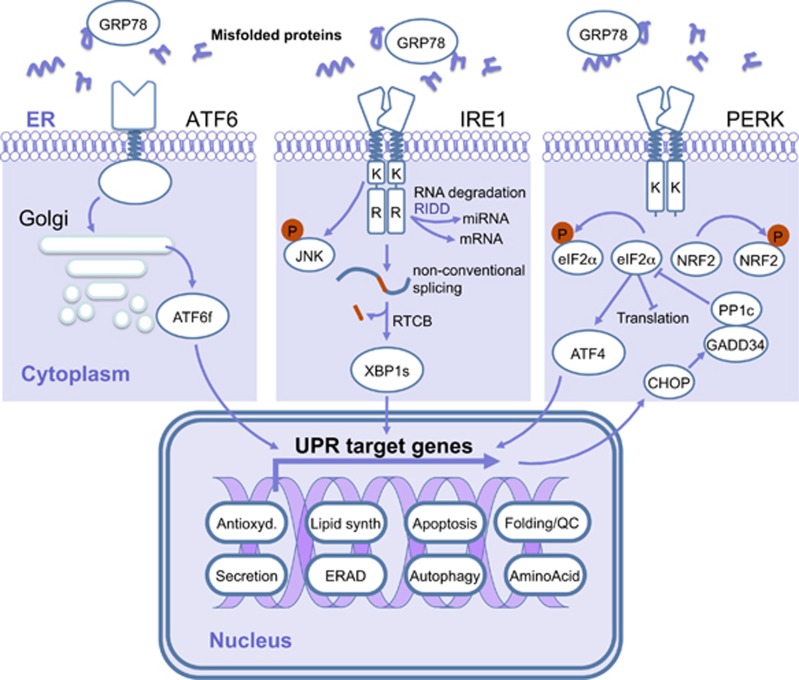
The UPR sensors and their downstream partners. During ER stress, GRP78 is released from IRE1α, PERK and ATF6 sensors allowing their dimerization/oligomerization or export to the Golgi apparatus. PERK activation leads to phosphorylation of NRF2 and eIF2α. Phosphorylation of eIF2α induces global translation attenuation and prompts that of AFT4. ATF4 and NRF2 induce expression of genes involved in antioxidant response, protein folding, amino-acid metabolism, autophagy and apoptosis. The negative feedback loop activated downstream of PERK dephosphorylates eIF2α to restore translation. IRE1α activation leads to c-Jun N-terminal protein kinase (JNK) phosphorylation, regulated IRE1-dependent decay (RIDD) activity and XBP1 splicing that induces expression of genes involved protein folding, secretion, ERAD and lipid synthesis. Activation of ATF6 leads to its export in the Golgi apparatus where its cytosolic domain is released to translocate to the nucleus and activate the transcription of genes involved in protein folding and ERAD. Antioxid, antioxidant response; Lipid synth, lipid synthesis; QC, quality control.

The three major mammalian UPR sensors were first described in the late 1990s: ATF6α (activating transcription factor 6α),^12^ IRE1α (inositol requiring enzyme 1α)^13^ and PERK (protein kinase RNA-activated-like ER kinase).^14^ The signaling pathways activated downstream of the three sensors lead to the reduction of protein misfolding, by slowing down *de novo* protein synthesis on the cytosolic side of the ER and by increasing protein folding and clearance in the ER (Figure 1). The activation of these three sensors is controlled by the ER-resident chaperone molecule GRP78/BiP (glucose-regulated protein 78/binding immunoglobulin protein). Indeed, under basal conditions, GRP78 constitutively associates with the luminal domains of the sensors through a noncanonical binding, thus preventing their activation.^1, 2^ Upon accumulation of misfolded proteins, GRP78 dissociates from the sensors when misfolded proteins accumulate in the ER, through mechanism depending on its substrate binding domain.^15^ This induces IRE1α and PERK oligomerization and autotransphosphorylation^16^ and the subsequent activation of the downstream signaling cascades. Moreover, BiP dissociation from AFT6α together with protein disulfide isomerase (PDI)-mediated disulfide bond modification^17, 18^ promotes ATF6α export to the Golgi complex.^19, 20^

#### Activating transcription factor 6α

ER stress leads to ATF6α export from the ER to the Golgi apparatus where ATF6α proteolytic cleavage by S1P and S2P proteases releases an active membrane-free form ATF6f, which therefore translocates to the nucleus and induces the transcription of genes mainly involved in protein folding and ERAD.^2, 3, 21, 22^

#### Inositol requiring enzyme 1α

IRE1α is a type I ER-resident transmembrane protein. Its cytoplasmic domain presents two distinct molecular activities: a serine/threonine kinase and an endoribonuclease (RNase), resembling RNaseL. Upon ER stress, IRE1α dimerizes/oligomerizes and its trans-autophosphorylation induces a conformational change leading to endoribonuclease activation.^1^ The first substrate described for IRE1α RNase was X-box binding protein-1 (XBP1) mRNA that is processed together with the t-RNA ligase RTCB (RNA 2′,3′-cyclic phosphate and 5′-OH ligase) leading to a non-conventional mRNA splicing.^23^ The resulting open reading frame is shifted and leads to the translation of a stable and active transcription factor, XBP1s.^24, 25^ XBP1s activate the expression of genes involved in protein folding, secretion, ERAD and lipid synthesis.^2, 26, 27^ IRE1α RNase is also involved in ER-localized mRNA, ribosomal RNA and microRNAs degradation.^28, 29, 30, 31, 32, 33, 34^ This activity is named regulated IRE1-dependent decay. Importantly, regulated IRE1-dependent decay selectivity is highly dependent on IRE1α oligomerization state and the cell type, the precise mechanisms of regulated IRE1-dependent decay activation are still debated.^35, 36, 37, 38^

#### PKR-like ER kinase

As for IRE1α, PERK is a type I ER-resident transmembrane protein. Upon ER stress, PERK trans-autophosphorylates and phosphorylates the translation initiation factor eIF2α (eukaryotic initiation factor 2α) and the transcription factor NRF2 (nuclear respiratory factor 2). Activated eIF2α attenuates global protein translation, reducing the folding demand on the ER^2, 3, 39, 40^ whereas activated NRF2 controls the antioxidant response.^2^ PERK-mediated eIF2α phosphorylation also triggers the translational activation of the transcription factor ATF4 that induces expression of genes involved in protein folding, amino-acid metabolism, autophagy and apoptosis^1, 2, 41, 42^ such as the apoptosis-related gene *CEBP* (CCAAT/enhancer-binding protein) homologous protein CHOP (CEBP homologous protein/growth arrest and DNA-damaged-inductible protein 153 (GADD153)) that impacts on the control of cell death/survival outputs upon ER stress.^43^ Moreover, PERK/eIF2α activation is negatively controlled by a feedback mechanism involving the protein GADD34 induced by this PERK pathway, which, in association with the phosphatase PP1c (protein phosphatase 1c), is responsible for the dephosphorylation of eIF2α.^44^

### UPR involvement in cancers

The role of ER stress signaling as a key actor in cancer development has been first proposed in 2004^8^ and is now largely accepted by both the scientific and medical communities.^45^ For instance, increased expression levels of major actors of the UPR such as IRE1α, unspliced and spliced XBP1, PERK and ATF6 were observed in tissues sections from a variety of human tumors including brain, breast, gastric, kidney, liver, lung and pancreatic cancers (Table 1).^46, 47, 48, 49, 50, 51, 52, 53, 54, 55, 56, 57, 58, 59, 60, 61, 62, 63, 64, 65, 66, 67^ Moreover, the chaperone GRP78 is also found overexpressed in many cancers^46, 47, 48, 49, 50, 51, 52, 54, 56, 57, 58, 59, 60, 61, 62, 64, 65, 66^ and is involved in the dissemination/metastasis of human tumors. GRP78 overexpression is associated with higher tumor grades and reduced patients’ survival.^48, 53, 57, 59, 61, 65, 67^ In experimental models including tumor cell lines and mouse tumor xenografts, GRP78 was also shown to have an important role in regulating cancer hallmarks (Table 2).^46, 47, 48, 51, 54, 55, 56, 57, 59, 60, 61, 65, 66, 68, 69, 70, 71, 72, 73^ For example, GRP78 regulates tumor cell proliferation and migration.^47, 59, 65^

Tumor progression is characterized by UPR activation induced by the challenging growth conditions associated with hypoxia and anticancers drugs.^52^ Furthermore, tumor cells develop specific metabolic processes to adapt to such environment,^74^ and examples of highly dynamic network between cancer cells’ adaptation and resistance to environmental stresses and UPR signaling pathways will be illustrated in the following section.

#### UPR linked to cancer initiation

In the normal gastrointestinal tract, a differential expression of GPR78 is observed and is lower in intestinal stem cells and higher in more differentiated transit amplifying cells.^75^ Interestingly, most of the colorectal cancers (CRCs) derive from transformed intestinal stem cell in which activation of the PERK/eIF2α axis is associated with the loss of stemness.^76^ This suggests that cancer initiation might be linked to ER stress in the gastrointestinal tract.^3^ Remarkably, in a colitis-associated cancer model, the IRE1α pathway appears to have an important role in mediating ER stress that induces intestinal stem cell expansion.^77^ Indeed, XBP1 loss in epithelial cells results in intestinal stem cell hyperproliferation, therefore promoting initiating phases of cancer development.^3^

#### UPR linked to tumor quiescence and aggressiveness

Cancer cells must cope with strict growth conditions forced by their intrinsic condition (oncogene expression) but also by the tumor environment including chemotherapy, nutrient starvation and *in vivo* microenvironmental challenges. They therefore develop adaptive mechanisms such as a metabolic resting state called quiescence/dormancy. Regulation of tumor cell dormancy has been associated with the activation of both ATF6α and PERK-eIF2α. Both pathways were identified as a survival factors for quiescent but not proliferative squamous carcinoma cells^78^ and under hypoxia,^79^ respectively. In triple-negative breast cancers, the IRE1α/XBP1s axis is found constitutively active, thereby conferring higher aggressiveness due to XBP1s-mediated hypoxia-inducible factor-1α activation.^80^ In glioblastoma (GBM), tumor migration/invasion is associated to aggressiveness. Interestingly, IRE1α endoribonuclease activity regulates the extracellular matrix protein SPARC (secreted protein acidic and rich in cysteine) itself involved in tumor invasion.^81^

#### UPR-linked ‘secretory switch’ in cancer cells

To sustain their own important metabolic demands and to adapt to their challenging environment, cancer cells reprogram their secretome and the associated secretory pathway needed to support tumor functions and necessary for cancer progression.^3, 82^ For instance, tumor invasion is facilitated by change in secreted extracellular matrix components and matrix metalloproteases.^83, 84^ Tumor cell proliferation and neoangiogenesis (see below) are sustained through the secretion of growth factors, cytokines and chemokines.^3^ As ER is the major site of protein production that also orchestrates their secretion, activation of the UPR strongly modulates tumor cells’ secretory switch during cancer development.

#### UPR linked to tumor epithelial-to-mesenchymal transition

Epithelial-to-mesenchymal transition (EMT) is a physiological process used by cancer cells to acquire critical oncogenic features such as migration/invasion, stemness and drug resistance.^3^ EMT is controlled by specific transcription factors involved in these cell functions and the UPR has been often involved in the expression of these transcription factors. For instance, in breast tumors, increased expression of XBP1s is observed in metastatic tumors, which correlates with the EMT inducer SNAIL (snail-related protein).^85^ LOXL2 (lysyl oxidase like 2)/GRP78 interaction in the ER also activates the IRE1-XBP1 signaling pathway thereby inducing the expression of several EMT-linked transcription factors including SNAI1 (snail family transcriptional repressor), SNAI2, ZEB2 (zinc-finger E-box-binding homeobox 2) and TCF3 (transcription factor 3).^69^ Moreover, the overexpression of the TWIST (twist-related protein) transcription factor correlates with PERK constitutive activation.^86^ The ‘secretory switch’ induced by UPR might also contribute to EMT.^86, 87, 88^ Indeed, overexpression of Serpin B3, a serine/cysteine protease inhibitor, is associated with chronic UPR induction leading to nuclear factor-κB activation and interleukin-6 production. This results in an EMT-like phenotype in mammary epithelial cells.^89^ In GBM, dominant-negative form of IRE1α modulates the expression molecules involved in extracellular matrix structures, angiogenesis and inflammatory chemokines, thus reflecting a mesenchymal drift.^90^

#### UPR-linked tumor angiogenesis

Expression of proangiogenic factors is affected by the UPR in cancer cells. For instance, vascular endothelial growth factor-A (VEGF-A), interleukin-1β and interleukin-6 are induced downstream of IRE1α signaling in GBM cells.^90, 91^ Moreover, IRE1α-mediated mRNA cleavage of the circadian gene *PERIOD1*,^92^ an important mediator of GBM infiltration, also supports tumor angiogenesis through the regulation of the CXCL3 chemokine.^90^ Furthermore, in response to hypoxia, VEGF is also upregulated by the PERK-ATF4 branch of the UPR to induce angiogenesis.^2, 3, 74, 93^ Interestingly, the UPR-regulated ER chaperone ORP150 (oxygen-regulated protein 150) controls tumor angiogenesis by promoting the secretion of VEGF in prostatic and glioma cancer cells.^94, 95^

#### UPR-linked tumor metabolic processes

Under nutrient deprivation, cancer cells adapt their metabolic demand in part through activation of the UPR. Downstream of IRE1α, XBP1s activates the expression of key enzymes of the hexosamine biosynthetic pathway that convert glucose to UDP-acetylglucosamine.^96, 97^ These are substrates for the O- and N-glycosylation of proteins, thereby improving global proteotasis. In addition, through hypoxia-inducible factor-1α activation, XBP1s also actively promotes glucose uptake in triple-negative breast cancer cells, which in turn upregulates the expression of several proteins involved in glycolytic processes including the glucose transporter 1.^98^

#### UPR linked to tumor autophagy

Autophagy is a cellular process that allows cancer cells to generate additional energy supplies through the selective or non-selective degradation of protein aggregates or damaged organelles. Under hypoxia, activation of the PERK/eIF2α/ATF4 pathway is protective for tumor cells through autophagy induction via LC3B (autophagy protein microtubule-associated protein 1 light chain 3b) and ATG5 (autophagy protein 5).^99, 100, 101^ Similarly, TNF receptor associated factor 2 (TRAF2)/IRE1α activates c-Jun N-terminal protein kinase that also induces autophagy.^102^

## Chemotherapy resistance induced by UPR

### General mechanisms of resistance to chemotherapy in cancer

**Figure 2:**
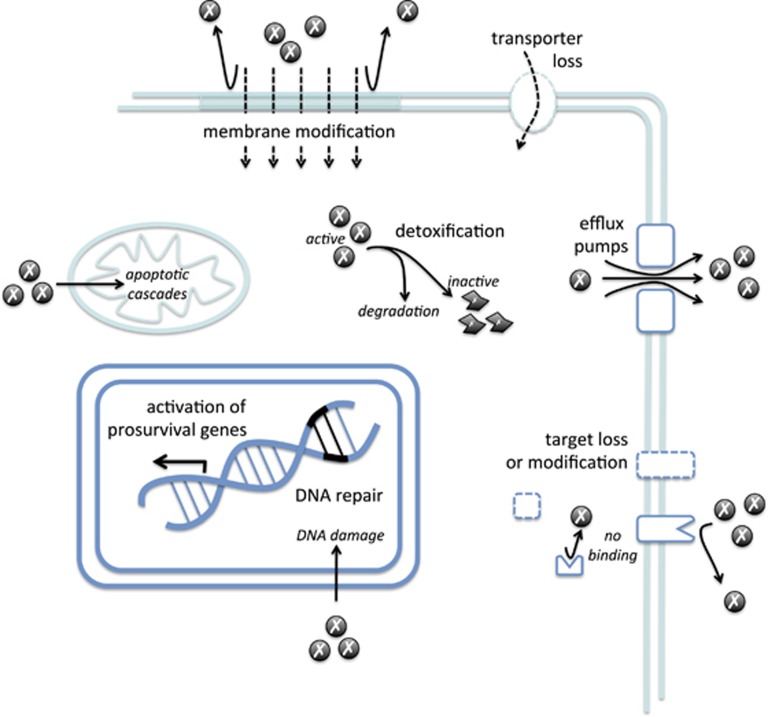
General mechanisms involved in chemotherapy resistance. Tumor cells limit chemotherapy drugs accumulation by modifying their membrane composition, reducing drug transporters and increasing efflux pumps. Mechanisms of detoxification lead to drug inactivation. Drug target modification or loss also contributes to chemotherapy resistance. Finally, DNA damage and apoptosis induced by anticancer drugs are prevented by sophisticated DNA repair system and upregulation of prosurvival genes.

During the past decades, chemotherapy and targeted therapies have become the principal modes of treatment against cancers (Table 3), but their efficacy is confronted to the multiple intrinsic and acquired resistance mechanisms developed by tumor cells before and during the treatment. These resistance mechanisms can include the reduction of drug uptake, the alteration of the drug target, the induction of drug-detoxifying mechanisms, repair of drug-induced damages and insensitivity to drug-induced cell death (Figure 2).^103, 104, 105^

#### Resistance to anticancer drug accumulation

Drugs enter into tumor cells by three main routes: diffusion, active transport and endocytosis.^103^ However, tumor cells use several mechanisms to limit this entry by decreasing the uptake or increasing the efflux of the drug.^103^ For instance, the family of multidrug resistance proteins, acting as drug efflux pumps (reviewed in Chen and Tiwari^106^and Sodani *et al.*^107^), is the subject of intense research to characterize the role in chemotherapy resistance.^11, 103^ Expression of these proteins has been reported to correlate with resistance to chemotherapy *in vitro*.^105^ Modulation of their functions is also correlated to *in vitro* chemosensitivity to drugs such as cisplatin, doxorubicin, paclitaxel and vincristine in several cancer cell lines.^108, 109^ In addition, modulation of the expression of cell surface transporters or their mutations can reduce drug uptake. As such, in osteosarcoma, both decreased expression and mutations of the methotrexate transporter reduced folate carrier that reduce their drug affinity have been reported.^103, 105, 110^ Finally, cancer cell mutants that have defective endocytosis are resistant to immunotoxins that enter into tumor cells by endocytosis.^103^

#### Induction of drug-detoxifying mechanisms

Both drug inactivation and the absence of drug activation are specific for given classes of drugs.^104^ For instance, 5-fluorouracil (5-FU) is catabolized by dihydropyrimidine dehydrogenase that confers *in vitro* resistance to 5-FU once overexpressed in CRCs.^105^ Platinum drugs such as cisplatin, carboplatin and oxaliplatin can also be inactivated after covalent linkage to the thiol glutathione, decreasing the availability of the native drug to bind its target^104, 108^ and leading to drug efflux by ABC transporter proteins.^105^ High levels of glutathione have been found in tumor cells resistant to platinum drugs. Interestingly, expression of glutathione S-transferase-π, a member of the family of glutathione S-transferase that catalyzes glutathione conjugation, is linked to overall survival following cisplatin treatment of head and neck cancers and to cisplatin resistance of ovarian cancers.^105, 108, 110^

#### Modification of drug targets

Drug sensitivity is affected by alterations of the drug target, such as mutations and/or changes in expression level.^104, 108^ For instance, 5-FU and pemetrexed treatments inhibit translation of their target mRNA thymidylate synthase (TS),^104^ thus leading to increased TS expression level and increased 5-FU resistance.^104, 105^ Moreover, the overexpression and/or oncogenic mutations in many protein tyrosine kinases have been described in human cancers, rendering difficult the anti-protein tyrosine kinase targeting therapies. Indeed, efficacy of epidermal growth factor receptor (EGFR) inhibitors such as gefitinib and erlotinib is markedly reduced in non-small-cell lung cancers exhibiting the EGFR-T790M mutation.^104^ Amplification and mutations in anaplastic lymphoma kinase have been identified in pediatric neuroblastoma, but secondary mutations in the anaplastic lymphoma kinase tyrosine kinase domain or anaplastic lymphoma kinase fusion gene amplifications are observed after crizotinib treatment leading to the disease relapse.^104^

#### DNA-damage repair

Most chemotherapeutic drugs drive the induction of DNA damage in tumor cells either directly for platinum-based drugs or indirectly for 5-FU and topoisomerase inhibitors.^104, 105^ DNA topoisomerase-I mutations have been reported to affect camptothecin sensitivity.^105^ Similarly, DNA topoisomerase-II, a target of doxorubicin and etoposide, is mutated in resistant cancer cell lines.^105^ Reduction of DNA topoisomerase-II expression by post-transcriptional modifications such as ubiquitination and sumoylation also leads to drug resistance and reduction of DNA damage.^6, 111^ In normal cells, DNA lesions are quickly recognized by DNA-damage response factors, which activate cell cycle checkpoints and direct DNA repair.^112^ Consequently, the regulation of DNA repair systems in tumor cells is a critical factor for their response to chemotherapeutics.^112^ For instance platinum-induced DNA damage is repaired by the nucleotide excision repair pathway and *in vitro* correlation between enhanced nucleotide excision repair and resistance to cisplatin has been reported in many studies.^108^ High expression of ERCC1 (excision repair cross-complementing 1), one of the key components of nucleotide excision repair, is linked to poor response to chemotherapy in numerous cancer types.^104^ In addition, mutation and/or downregulation of key DNA mismatch repair proteins such as MLH1 (mutL homolog 1) is observed in cisplatin-resistant tumors.^104, 108, 110^

#### Activation of antiapoptotic and prosurvival pathways

Most tumors develop defects in the common cell death pathways that lead to chemotherapy resistance.^104^ For instance, levels of BIM (Bcl-2 interacting mediator of cell death), a proapoptotic protein of the Bcl-2 (B-cell lymphoma) family, predict clinical responsiveness to EGFR and ERBB2 inhibitors. Moreover, a germline deletion in *BIM* gene is significantly associated with resistance to protein tyrosine kinase inhibitors in patients with EGFR-mutant lung cancers.^104^ Expression levels of MCL1, another member of the Bcl-2 family, are important determinant of resistance to Bcl-2 inhibitor ABT-737 and other cytotoxic chemotherapeutics.^104^ Furthermore, under chemotherapy pressure, tumors develop novel survival signaling pathways that contribute to drug resistance.^104^ An important number of proteins is involved in these pathways: oncogenes such as *RAS* and *AKT* (v-Akt murine thymoma viral oncogene homolog); tumor suppressor genes such as *TP53* (tumor protein 53) and *PTEN* (phosphatase and tensin homolog); and prosurvival factors as nuclear factor-κB and signal transducer and activator of transcription 3.^104, 108^ Mutations, amplifications, chromosomal translocations and overexpression of these genes are associated with various malignancies and linked to resistance to chemotherapy and targeted therapies.^104^

#### Other factors involved in drug resistance

The influence of the local tumor microenvironment is identified as important contributor to chemotherapy resistance.^104^ For instance, hypoxia enhances drug detoxification by interfering with the generation of oxygen radicals and by increasing hypoxia-inducible factor-1-mediated activation of survival signals.^108^ Furthermore tumor heterogeneity at the genetic, molecular and cellular levels contributes substantially to chemotherapy resistance. For instance, the presence of cancer stem cells with robust intrinsic drug resistance capabilities reduces the chemotherapy efficacy.^104^ In solid tumors, the stroma (extracellular matrix, cancer-associated fibroblasts, immune and inflammatory cells and blood vessels) protects cancer cells from cytotoxic agents, thus allowing them to evade apoptosis and to develop acquired resistance leading to disease relapse.^11, 104, 108^ Recently, EMT has been associated with chemotherapy and targeted therapy resistance.^104^ Finally, as most anticancer drugs are primarily targeted against proliferating cancer cells, a significant proportion of cancer cells are in a dormancy/quiescent state, thereby exhibiting a degree of drug resistance linked to their decreased ability to proliferate.^11, 108^

### Chemotherapy resistance induced by the UPR

**Figure 3:**
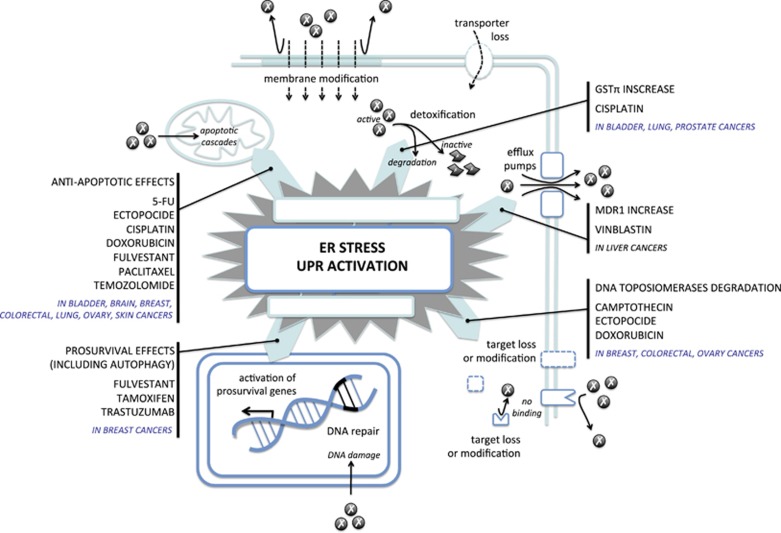
The UPR intervention in chemotherapy resistance. UPR activation contributes to chemotherapy drug resistance by increasing drug detoxification and efflux pump expression, by modulating drug targets and activating antiapoptotic and prosurvival genes expression. Examples of anticancer drugs used several cancer types described in the literature are indicated.

UPR activation is commonly observed in various tumor specimens (see UPR involvement in cancers) and correlates with drug resistance. Clinical evidences and *in vitro* demonstrations of tight link between UPR activation and drug resistance will be first reviewed in this section. The link between UPR and cellular adaptation of cancer cells including autophagy and hypoxia that also contributes to antidrug resistance will be presented in the next paragraphs (Figure 3).

#### Clinical relevance of the UPR activation and chemotherapy resistance

Clinical evidences of such phenomenon are almost exclusively limited to breast cancers (Table 4).^49, 52, 113, 114, 115^ Indeed, expression of the UPR sensors and their downstream partners are correlated with resistance to tamoxifen, thereby leading to decreased time to recurrence and poor survival.^52^ Interestingly, opposite effects are observed with the expression of XBP1u and XBP1s. XBP1u is associated with longer survival of breast patients treated with tamoxifen, whereas XBP1s is associated with shorter survival.^113^ This underlines IRE1α involvement in tamoxifen resistance. In contrast, GRP78 involvement seems to be more complex. High GRP78 expression in breast cancer specimens predicts a shorter recurrence-free survival in patients who received doxorubicin-based adjuvant chemotherapy. However, the opposite effect is observed in patients treated with doxorubicin and cyclophosphamide, followed by taxane (paclitaxel or docetaxel) on a clinical trial, where GRP78-positive staining predicts a better recurrence-free survival.^114^ These results underline the possibility of use combined anticancer drugs to overcome cancer resistance (Figure 3).

#### Induction of UPR-dependent chemotherapy resistance *in vitro*

Correlations between UPR activation and chemotherapy resistance were mainly demonstrated in cellular models in many types of cancer (Table 5).^46, 47, 48, 51, 52, 53, 54, 57, 60, 62, 64, 71, 72, 116, 117, 118, 119, 120, 121, 122, 123, 124, 125, 126, 127, 128, 129, 130^ A vast number of these studies demonstrate the impact of GRP78 expression on drug resistance mainly involving a reduced effect of drug-induced apoptosis.^47, 48, 54, 60, 64, 116, 117, 120, 123, 125, 128, 129^ However, the precise molecular mechanisms involved remain to be discovered. In chemotherapy-resistant breast cancer cells, GRP78 suppresses doxorubicin-mediated apoptosis in part through inhibition of BAX (Bcl-2-associated X protein) and caspase-7 activation.^49^ GRP78 also forms complexes with BIK (Bcl-2-interacting killer), an apoptotic BH3-only protein, and blocks its apoptotic activity under estrogen starvation.^120^ Finally, the PDIA5/ATF6α activation loop was described to be essential to confer imatinib resistance in K562 leukemia cells.^17^ The direct involvement of the UPR sensors in other mechanisms associated with cancer resistance to chemotherapy (i.e. reduction of anticancer drug accumulation, drug-detoxifying mechanisms, modification of drug targets and DNA-damage repair) is up to now rather limited. For instance, a role for PERK in chemotherapy-resistant HT29 colon cancer cells has been involved in the upregulation of MDR related protein 1 through the regulation of NRF2.^131^

#### UPR and cellular adaptation links to cancer chemotherapy resistance

Different anticancer treatments, including those that stimulate ER stress, activate autophagy in tumor cells, which has been proposed to either enhance cancer cell death or act as a mechanism of resistance to chemotherapy.^104, 132^ Indeed, autophagy is a lysosome-dependent degradation pathway that degrades cellular components to maintain cellular biosynthesis and viability during metabolic stresses such as nutrient deprivation. During chemotherapy, autophagy facilitates cancer cell survival to cope with metabolic stresses caused by anticancer drugs.^104^ For instance, in breast cancer cell models, resistance to endocrine therapy such as tamoxifen and fulvestrant is the result of activation and interactions between different cellular mechanisms including UPR activation, autophagy and apoptosis in breast cancers.^122, 123, 125, 126, 133^ Indeed, antiestrogen-resistant breast cancer cells display higher levels of basal autophagy than sensitive cells.^123^ In addition, XBP1s-overexpressing MCF-7 cells displayed much higher basal levels of autophagy as demonstrated with increased basal LC3II levels and decreased p62 levels.^123^ Autophagy induced by XBP1s overexpression protects the cells against apoptosis. Furthermore, XBP1s-overexpressing cells become sensitive to tamoxifen when autophagy is blocked.^123^

Hypoxia is known to confer cancer cells with resistance to chemotherapy and to modulate UPR during ER stress.^134, 135, 136^ In breast cancers, taxol rapidly induces UPR activation including ATF6α, IRE1α and PERK pathways. However, hypoxia modulates taxol-induced UPR activation acting specifically on the UPR branches PERK, ATF6α and IRE1α.^137^ Indeed, ATF4 activation leads to taxol-induced autophagy completion and cell death resistance. Finally, ATF4 expression in association with hypoxia-induced genes, such as adrenomedullin, is a biomarker of a poor prognosis for human breast cancer patients.^137^ Intratumoral hypoxia is one predominant feature of GBM and is associated with resistance to temozolomide (TMZ), the standard chemotherapy for GBM.^138^ TMZ sensitivity of both sensitive and resistant GBM cells is significantly enhanced under hyperoxia *in vitro* through the induction of caspase-dependent pathways.^138^ In addition, elevated PDIA1 expression also occurs in hypoxic brain tumor cells. PDIA1, which belongs to the protein disulfide isomerase superfamily, is the key foldase that has been found to be significantly dysregulated during the development of TMZ resistance in GBM cells.^139^ Hyperoxia resensitizes TMZ-resistant GBM cells to TMZ by abrogating the hypoxia-induced UPR-related protective mechanisms. Hyperoxia, alone or synergistically with TMZ, activates the UPR in sensitive and resistant cell lines.^139^ Hyperoxia impairs protein folding that in turn induces UPR-mediated apoptosis. Its reduces survival benefit of cancer cells with PDIA1 overexpression through the UPR by decreasing GRP78 and PDIA1 expression and consequently triggering cell death via downregulation of the ER stress chaperone protectors.^139^ Interestingly, TMZ increases galectin-1 expression in glioma cells.^134^ Galectin-1 increases the expression of genes implicated in chemotherapy resistance such as *GRP78*, *ORP150*, *HERP* (homocysteine-induced ER protein), transcription associated factor 1 (TRA1), *BNIP3L* (Bcl-2/adenovirus E1B 19 kDa protein-interacting protein 3-like), *GADD45*B and *CYR61* (cysteine-rich angiogenic inducer 61), some of which are located in the ER and modified by hypoxia.^134^ Additionally, under severe hypoxia and chemotherapy, UPR activation occurs in hypopharyngeal carcinomas leading to increased expression of GRP78 associated with hypoxia-induced chemotherapy resistance.^136^ Diminution of GRP78 inhibits cell proliferation and promotes apoptosis under cisplatin treatment with severely hypoxic conditions, indicating that GRP78 confers cancer cell resistance to cisplatin in response to severe hypoxia. This phenomenon involves increased CHOP and BAX expression levels and decreased Bcl-2 expression levels with simultaneous increased apoptosis under severely hypoxic conditions.^136^ A number of studies indicated that improving oxygenation inside the tumor could serve as a potential strategy to target hypoxia-induced chemotherapy resistance.^135^ In liver cancers, hypoxia increases cisplatin resistance. The use of a hemoglobin-based oxygen carrier (OC89) enhances the efficacy of cisplatin-based transarterial chemoembolization in rat liver cancer model. OC89 delivery knocks down the balance of UPR pathway by decreasing GRP78 expression and increasing that of CHOP. This leads to increase tumor apoptosis and to inhibit tumor cell proliferation.^135^

Interestingly, UPR activation is also observed in non-tumoral cells that compose the tumor microenvironment.^140^ Indeed, UPR markers GRP78, ATF4 and CHOP are significantly upregulated in endothelial cells from oral squamous cell carcinomas. Furthermore, under severe acidic conditions and hypoxia, which recapitulate the tumor microenvironment, microvascular endothelial cells increase GRP78 expression, acquire antiapoptosis capacities and resist to sunitinib, an antiangiogenic drug.^140^ GRP78 knockdown resensitizes endothelial cells to drug treatment.^140^

## Conclusion and perspectives: targeting the UPR to bypass resistance

The UPR is a physiological mechanism developed by cells to cope with misfolded protein accumulation induced by challenging conditions. As observed for other cellular mechanisms, tumor cells hijack the UPR to allow drug resistance, through the activation of the UPR sensors ATF6, IRE1α and PERK, and their master regulator GRP78. As presented above, the involvement of the UPR in chemotherapy resistance is complex and not fully covered yet. This is in part due to the links between the UPR and other tumor adaptive mechanisms as such antiapoptotic mechanisms, autophagy or dormancy. Therefore, a global understanding of the molecular mechanisms controlling UPR-mediated drug resistance is highly needed.

Small-molecule UPR inhibitors that directly target the UPR sensors ATF6α, IRE1α, PERK and their regulators or effectors such as PDIA1 and eIF2α, respectively, have been recently identified.^141^ Their potential use in combination with chemotherapeutics might greatly improve anticancer drug efficacy. For instance, ISRIB, a drug that reverses the effects of eIF2α phosphorylation, increased gemcitabine-induced death of pancreatic cancer cells.^142^ Recent evidences have also been provided from leukemic tumors. The PDI inhibitor 16F16 reverses leukemia cell resistance to imatinib linked to the ATF6α pathway most likely by blocking PDIA5.^17^ Finally, MKC-3946, an IRE1α RNase inhibitor, synergizes bortezomib or arsenic trioxide induced toxicity of acute myeloid leukemia cells.^143^

Alternatively, modulating UPR with pharmacological drugs has shown promising results *in vitro*. For instance, epigallocatechin gallate, which specifically targets GRP78, resensitizes glioma cells to TMZ.^47, 144^ Although targeting GRP78 might be an attractive therapeutic approach, the challenge will be to minimize systemic toxicity in normal organs in which GRP78 is essential for the survival and functions of various cellular subtypes.^145^ This implies that GRP78-targeting drugs should selectively target tumor cells that require a high level of GRP78 and spare normal organs. Bortezomib, a proteasome inhibitor that amplifies the protein misfolding burden, confers a chemosensitizing effect to cisplatin, doxorubicin or camptothecin in various tumor types including breast, colon pancreatic cancers.^146^ Sorafenib, a potent multikinase inhibitor, induces both apoptosis and autophagy in human hepatocellular carcinoma cells through an ER stress-dependent mechanism and the alteration of normal secretory functions. Furthermore, the combination of sorafenib with the autophagy inhibitor chloroquine leads to enhance liver cancer suppression.^147^ Verteporfin, a YAP1 (Yes-associated protein 1) inhibitor, has been recently involved in the oligomerized protein accumulation in CRC cells, leading in part to tumor apoptosis. Furthermore, hypoxic or nutrient-deprived conditions amplify verteporfin-mediated CRC cell death.^148^ Resistance of melanoma cells to vemurafenib or PLX4032, two BRAFV600E kinase inhibitors, is bypassed in the presence of thapsigargin, an inhibitor of the SERCA pumps or in the presence of HA15, which targets GRP78, respectively, by inducing tumor apoptosis.^73, 149^

In conclusion, future challenges will certainly lead to the development of combined therapeutic approaches with new drugs that specifically target the UPR sensors and downstream partners and will to bypass anticancer drug resistance.

## Figures and Tables

**Table 1:** Clinical evidences of UPR involvement in solid cancers

*Tumor origin*	*Materials*	*Methods*	*GRP78*	*IRE1α*	*XBP1*	*XBP1s*	*ATF6*	*PERK*	*eIF2α*	*Others*	*Comments*	*Ref.*
Brain	GBM	IHC, WB	+		+	+	+			(1)		^46^
	GBM	WB	+									^47^
	GBM, AAIII, AAII, ODG	Transcriptomic, IHC, WB	+								Increased in high-grade tumors	^48^
												
Breast	Invasive (stages II and III)	IHC	+									^49^
	Ductal, lobular, stages II and III	NB, IHC, WB	+									^50^
	adenocarcinoma	IHC	+		+						Correlated with ERα expression	^51^
	ERα+ invasive ductal carcinoma	transcriptomic	+	+	+		+		**+**	(2)		^52^
	ERα+	IHC				+					Associated with poor prognosis	^53^
												
Colorectal	stages II and III CRC	IHC	+									^54^
	Adenoma, CRC	RT–PCR, IHC			+							^55^
	CRC	IHC	+								No correlation with grade or metastases	^56^
	CRC	IHC	+								Increased in metastatic tumors	^57^
	Adenoma, adenocarcinoma	IHC	+							(3)		^58^
												
Kidney	RCC (stages I– IV)	Q-PCR, IHC	+								Associated with high-stage tumors	^59^
												
Liver	HCC	IHC	+									^60^
	HCC	NB, Q-PCR, IHC	+		+	+	+				Associated with histologic grading	^61^
	HCC	IHC	+								Correlated with CD147 expression	^62^
												
Lung	Adenocarcinoma	Q-PCR		+			+	+		(4)	Associated with low stages	^63^
	NSCLC	IHC	+								Correlated with RRBP1 expression	^64^

Pancreas	PDAC	IHC	+								Associated with poor prognosis	^65^
	PDAC	RT–PCR, IHC	+	+	+		+	+		(5)	Associated with MIA2 mutations	^66^
	PDAC	IHC								(6)	Associated with poor prognossis correlated with decreased SMARCB1 expression	^67^

Abbreviations: AA, anaplastic astrocytoma; ATF, activating transcription factor; CRC, colorectal cancer; eIF2α, eukaryotic initiation factor 2α ERp, ER protein; GADD, growth arrest and DNA-damage-inducible protein; GBM, glioblastoma; HCC, hepatocellular carcinoma; IRE1α, inositol requiring enzyme 1α GRP, glucose-regulated protein; IHC, immunohistochemistry; NB, northern blot; NSCLC, non-small cell lung cancer; ODG, oligodendroglioma; PCR, polymerase chain reaction; PDAC, pancreatic ductal adenocarcinoma; PDI, protein disulfide isomerase; PERK, PKR-like endoplasmic reticulum kinase; Q-PCR, quantitative PCR; RCC, renal cell carcinoma; RT–PCR, reverse transcriptase–PCR; SERP, stress-associated ER protein; UPR, unfolded protein response; WB, western blot; XBP, X-box binding protein.

(1) Calreticulin(+), CHOP/GADD153(+), ERp72(+), GRP94(+), GRP170(+).

(2) CHOP(+), GADD34(+), GRP94(+), SERP1(+).

(3) Decreased CHOP.

(4) ERO1A.

(5) Calnexin(+), PDI(+).

(6) Phosphorylated ATF2.

**Table 2:** Cellular models demonstrating the importance of UPR in solid cancers

*Tumor origin*	*Materials*	*Methods*	*GRP78*	*IRE1α*	*XBP1*	*XBP1s*	*ATF6*	*PERK*	*eIF2α*	*ATF4*	*Others*	*Comments*	*Ref.*
Brain	U87 cell line	NB, WB	+								(1)		^46^
	U87 xenograft	NB, IHC, WB	+				+			+	(2)		
	U87 and D245MG xenografts	NB, IHC, WB	+		+					+	(3)		
	U87, U251, U138, A172, LN229 and T98G	WB, IHC	+									Associated with increased proliferation	^47^
	U87, U251, A172, LN229, LN443 and LNZ308	WB	+										^48^
	U251	RT–PCR	+	+		+	+			+	(4)	Increased under arginine deprivation	^68^
													
Breast	T47D cell line	WB	+									Increased under glucose privation increased under estrogen treatment	^51^
	Hs578T, MDA-MB-231		+	+		+		+	+		(5)	Modulated by LOXL2 and associated with EMT	^69^
													
Colorectal	Colo205, HCT116, SW480, SW626	RT–PCR, WB	+	+	+	+	+	+	+		(6)		^54^
	DLD1, HCT15, SW480, WiDr	RT–PCR			+								^55^
	Colo205, HCT116, SW480, SW626	RT–PCR, WB	+		+	+	+	+	+		(7)		^57^
	HT29	WB	+									Increased under glucose deprivation or radiation	^56^
	HCT119	RT–PCR, WB	+	+		+	+			+	(8)	Increased under arginine deprivation	^68^
	HT29	RT–PCR, WB	+	+		+	+			+	(9)		
	HGC27	WB					+	+		+		Increased under severe hypoxia	^70^
													
Kidney	786-O, OS-RC-2 and Caki-1	RT–PCR, WB	+										^59^
	786-O, A498, ACHN, Caki,	RT–PCR, WB	+									Associated with increased proliferation	^71^
													
Liver	HepG2	WB	+									Increased under glucose privation	^60^
	HepG2, HuH7, HLF	NB, WB	+		+	+	+						^61^
	HepG2, SMCC-7721, MHCC97-H	WB	+	+							(10)		^72^
													
Ovary	SKOV3	RT–PCR	+	+		+				+	(11)	Increased under arginine deprivation	^68^
													
Pancreas	AsPC-1, BxPC-3, Capan-1, MIAPaCa-2, PCT-3 and SU.86.86	WB	+									Associated with increased proliferation and migration	^65^
	Su86.86	RT–PCR		+								Associated with MIA2 mutations	^66^
													
Skin	A375, HMVII, WM9, WM3918	RT–PCR, WB		+	+	+		+	+	+	(12)	Increased by HA15, a GRP78 inhibitor	^73^

Abbreviations: ATF, activating transcription factor; EDEM, ER degradation enhancer, mannosidase α-like; eIF2α, eukaryotic initiation factor 2α EMT, epithelial-to-mesenchymal transition; ERp, ER protein; GRP, glucose-regulated protein; HERP, homocysteine-induced ER protein; IHC, immunohistochemistry; IRE1α, inositol requiring enzyme 1α LOXL2, lysyl oxidase like 2; NB, northern blot; PDI, protein disulfide isomerase; PERK, PKR-like endoplasmic reticulum kinase; UPR, unfolded protein response; WB, western blot; XBP, X-box binding protein.

(1) GRP94(+).

(2) CHOP(+).

(3) Calreticulin(+), CHOP(+), ERp72(+), GRP94(+), HERP(+), PDI(+).

(4) CHOP(+), EDEM1(+), GRP94(+).

(5) DDIT3(+), DNAJB9(+), EDEM1(+).

(6) Phosphorylated PERK and eIF2α.

(7) Phosphorylated eIF2α.

(8) CHOP(+), GRP94(+), phosphorylated eIF2α and GCN2.

(9) CHOP(+), EDEM1(+), phosphorylated eIF2α and GCN2.

(10) Phosphorylated IRE1α.

(11) CHOP+, GRP94+.

(12) CHOP(+), phosphorylated IRE1α, PERK and eIF2α.

**Table 3:** Standard chemotherapy treatments and their targets in solid tumors

*Drugs*	*Cancers*	*Targets*
*Alkylating agents*		
Carboplatin	Ovary	DNA alkylation
Cisplatin	Biliary, gastric, lung, urogenital	DNA alkylation
Cyclophosphamide	Urinary	DNA alkylation
Dacarbazine	Skin	DNA alkylation
Ifosfamide	Soft tissues	Guanine alkylation
Oxaliplatin	Biliary, colorectal, pancreas	DNA crosslinking
Temozolomide	Brain	Guanine alkylation
		
*Antimetabolites*		
5-Fluorouracil	Colorectal, gastric, pancreas	Pyrimidine analog, TS
Capecitabine	Breast, colorectal	Pyrimidine analog, TS
Gemcitabine	Biliary, lung, pancreas, urinary	Deoxycytidine analog
Methotrexate	Urinary	DHFR
Pemetrexed	Lung	TS, DHFR, GARFT
		
*Antibiotics/intercalants*		
Doxorubicin	Endometrial, soft tissues, urinary	DNA intercalant
Camptothecin	Colorectal, pancreas	Topoisomerases I
Etoposide	Lung, urogenital	Topoisomerases II
Bleomycin	Genitourinary	DNA strand break inducer
		
*Antimitotics/spindle poisons*		
Docetaxel	Breast, gastric, urinary	β-Tubulin
Paclitaxel	Breast, ovary	β-Tubulin
Vinblastin	Breast, kidney, urinary	Microtubules
		
*Hormone therapy*		
Bicalutamide	Prostate	Androgen receptors
Goserelin	Prostate	GnRH agonist
Tamoxifen	Breast	Estrogen receptors
		
*Targeted therapy*		
Erlotinib	Pancreas	EGFR
Bortezomib	*Lymphoma, myeloma*	Proteasome
Sorafenib	Kidney, liver	FLT3, c-KIT, PDFGRβ, c-RAF, b-RAF, VEGFRII and III
Sunitinib	Kidney	FLT3, c-KIT, PDGFRβ, RET, VEGFRI and II
		
*Immunotherapy*		
Bevacizumab	Kidney, lung	VEGF
Trastuzumab	Breast	HER2/neu

Abbreviations: DHFR, dihydrofolate reductase; EGFR, epidermal growth factor receptor; FLT, fms-like tyrosine kinase; GARFT, glycinamide ribonucleotide formyltransferase; GnRH, gonadotropin-releasing hormone; HER2/neu, human epidermal growth factor receptor; KIT, v-kit Hardy-Zuckerman 4 feline sarcoma viral oncogene homolog; PDGFR, platelet-derived growth factor receptor; RAF, rapidly accelerated fibrosarcoma; RET, rearranged during transfection; TS, thymidylate synthase.

**Table 4:** Clinical evidences of UPR involvement in cancer chemotherapy resistance

*Tumor origin*	*Materials*	*Chemotherapy*	*Methods*	*GRP78*	*IRE1α*	*XBP1*	*XBP1s*	*ATF6*	*PERK*	*Others*	*Comments*	*Ref.*
Breast	Ductal/lobular (stages II and III)	Doxorubicin	IHC	+							Associated with reduced time to recurrence	^49^
	ERα+	Tamoxifen	Transcriptomic		+			+	+	(1)	Associated with poor prognosis	^52^
	Invasive ductal (stages I–III)	Tamoxifen	Q-PCR			+	+				Associated with high or poor survival respectively	^113^
	Invasive ductal (stages II and III)	Doxorubicin, cyclophosphamide+ taxane (paclitaxel or docetaxel)	IHC	+							Associated with longer survival	^114^
												
Colorectal	Rectal cancer	5-FU	WB							(2)	Associated with poor response to therapy	^115^

Abbreviations: ATF, activating transcription factor; eIF2α, eukaryotic initiation factor 2α ER, estrogen receptor; ERO1L, ER oxidoreduction 1-like; 5-FU, 5-fluorouracil; GADD, growth arrest and DNA-damage-inducible protein; GRP, glucose-regulated protein; HERPUD, HERP ubiquitin-like domain; IHC, immunohistochemistry; IRE1α, inositol requiring enzyme 1α PERK, PKR-like endoplasmic reticulum kinase; Q-PCR, quantitative PCR; RT–PCR, reverse transcriptase–PCR; SERP1, stress-associated ER protein 1; SYNV, synoviolin; UPR, unfolded protein response; XBP, X-box binding protein.

(1) 18 genes: *ATF4*, *ATF6α*, *CHOP*, *DNAJB9*, *DNAJC3*, *EDEM1*, *eIF2α*, *ERO1L*, *ERO1LB*, *GADD34*, *GRP78*, *GRP94*, *HERPUD1*, *IRE1α*, *PERK*, *XBP1*, *SERP1*, *SYNV1*.

(2) Calnexin(+).

**Table 5:** Cellular models demonstrating the importance of UPR in cancer chemotherapy resistance

*Tumor origin*	*Materials*	*Chemotherapy*	*Methods*	*GRP78*	*IRE1α*	*XBP1*	*XBP1s*	*ATF6*	*PERK*	*eIF2α*	*ATF4*	*Others*	*Comments*	*Ref.*
Bladder	T24/83	Etoposide, doxorubicin, camptothecin	WB	+									Associated with resistance to apoptosis	^116^
														
Bone	MG-63, SaOS-2	Cisplatin	WB	+								(1)	Associated with resistance to apoptosis	^117^
														
Brain	U87	Temozolomide	WB	+									Increased with ER stress (DTT)	^46^
	U87 and U251	Temozolomide	WB	+								(1)		^47^
	LN229	Temozolomide, camptothecin, 5-FU	WB	+									Associated with resistance to apoptosis	^47^
	A172 and LNZ308	Etoposide, cisplatin	IHC	+									Associated with resistance to apoptosis	^48^
	U87 and U251	Temozolomide						+			+	(2)	Associated with radicol-induced apoptosis	^118^
														
Breast	MCF-7	Doxorubicin	WB	+								(3)		^119^
	T47D	Estrogen	Q-PCR, WB	+			+				+	(4)		^52^
	MCF-7	Estrogen	Q-PCR, WB	+	+	+	+	+	+	+		(5)		^52^
	MCF-7 xenograft	Estrogen	Q-PCR	+	−	−	+	−	−	+		(6)		^52^
	293T, MCF-7	Etoposide	WB	+									Associated with BIK interaction	^120^
	MCF-7, T47D	Fulvestrant	WB			+	+							^121^
	LCC1, LCC9	Fulvestrant	WB	+			+				+	(7)	Associated with autophagy	^122^
	LCC9, MCF-7	Fulvestrant	WB			+	+						Associated with resistance to apoptosis	^123^
	MDA-M35, T47D, MCF-7	Quercetin	Q-PCR, WB	+				+				(8)		^124^
	MCF-7	Paclitaxel	WB	+									Associated with resistance to apoptosis	^125^
	T47D	Tamoxifen	WB	+										^51^
	MCF-7, T47D MCF-7 xenograft	Tamoxifen Tamoxifen	RT–PCR, WB WB				+ +					(9)	Decreased resistance with IRE1 inhibitor decreased with IRE1 inhibitor	^53^
	MCF-7, T47D	Tamoxifen	WB			+	+							^121^
	MCF-7 xenograft	Tamoxifen	WB			+	+							^121^
	Rat DMBA-induced mammary tumors	Tamoxifen	WB	+	+						+	(1)	Associated with autophagy	^126^
	SKBr3	Trastuzumab	Q-PCR, ELISA	+								(10)	Increased with ER stress (Tg)	^127^
Cervix	SiHa-derived stem-like cells	Cisplatin	RT–PCR, WB	+			+					(11)	increased apoptosis with IRE1 inhibitor	^128^
														
Colorectal	Colo205, HCT116, SW480, SW626	Cisplatin, 5-FU	WB	+									Associated with resistance to apoptosis	^54^
	HCT116 HT29	5-FU										(12)	Associated with resistance to apoptosis	^57^
														
Kidney	A498, ACHN	Doxorubicin, 5-FU	IHC	+									Associated to cell cycle control	^71^
														
Liver	HepG2	Doxorubicin	RT–PCR, WB	+									Increased survival under glucose privation	^60^
	7741, HepG2 and 7741 xenograft	Doxorubicin, VP-16	IHC, WB	+									Correlated with CD147 expression	^62^
	HepG2, MHCC97	Sorafenib					+					(9)	Associated with resistance to apoptosis-dependent of RACK expression	^72^
														
Lung	PC13, PC14	Doxorubicin	WB	+									Associated with resistance to apoptosis	^64^
														
Ovary	PEO4	Estrogen	Q-PCR, WB	+			+							^52^
	OVCAR-3	Paclitaxel	WB	+									Associated with resistance to apoptosis	^125^
														
Skin	Hep3 (dormant versus tumorigene)	Etoposide, doxorubicin	Q-PCR, WB	+								(13)	Associated with resistance to apoptosis	^129^
														
Others	CHO *(hamster)*	Etoposide, doxorubicin, camptothecin	WB	+									Associated with resistance to apoptosis	^116^
	CHO (*hamster***)**	Etoposide	WB	+									Increased under ER stress (tg)	^130^
	NIH3T3	Etoposide	WB	+									Increased under ER stress (tg)	^130^

Abbreviations: ATF, activating transcription factor; BIK, Bcl-2-interacting killer; DTT, dithiothreitol; eIF2α, eukaryotic initiation factor 2α ERO1L, ER oxidoreduction 1-like; 5-FU, 5-fluorouracil; FRP, glucose-regulated protein; HSP, heat-shock protein; IHC, immunohistochemistry; IRE1α, inositol requiring enzyme 1α JNK, c-Jun N-terminal protein kinase; LCN2, lipocalin 2; PDI, protein disulfide isomerase; PERK, PKR-like endoplasmic reticulum kinase; Q-PCR, quantitative PCR; RT–PCR, reverse transcriptase–PCR; Tg, thapsigargin; UPR, unfolded protein response; WB, western blot; XBP, X-box binding protein.

(1) CHOP(+).

(2) calnexin(+), calreticulin(+), CHOP(+), GRP94(+), PDI(−), phosphorylated IRE1α, PERK and eIF2α(+).

(3) CHOP(+), phosphorylated PERK.

(4) Decreased CHOP, cleaved ATF6, phosphorylated PERK and eIF2α.

(5) DNAJC3, ERO1LB, GRP94.

(6) CHOP(+), DNAJC3(−), ERO1Lb(−), GADD34(+).

(7) CHOP(+), GRP94(+), cleaved ATF6, phosphorylated eIF2α.

(8) CHOP(+), phosphorylated eIF2α and JNK.

(9) Phosphorylated IRE1α.

(10) CHOP(+), LCN2(+).

(11) Phosphorylated eIF2α.

(12) Calnexin(+).

(13) HSP47(+), PDI(+), phosphorylated PERK and eIF2.
